# Associations between biological markers of prenatal stress and infant negative emotionality are specific to sex

**DOI:** 10.1016/j.psyneuen.2017.09.004

**Published:** 2017-12

**Authors:** Elizabeth C. Braithwaite, Susannah E. Murphy, Paul G. Ramchandani, Jonathan Hill

**Affiliations:** a School of Psychology and Clinical Language Sciences, University of Reading, Reading, UK; b Department of Experimental Psychology, University of Oxford, 9 South Parks Road, Oxford, UK; c Department of Psychiatry, University of Oxford, Warneford Hospital, Oxford, UK; d The Centre for Psychiatry, Imperial College London, Hammersmith Campus, Du Cane Road, London, W12 ONN, UK

**Keywords:** Prenatal stress, Glucocorticoids, Fetal programming, Alpha-amylase

## Abstract

**Purpose:**

Fetal programming is the idea that environmental stimuli can alter the development of the fetus, which may have a long-term effect on the child. We have recently reported that maternal prenatal cortisol predicts infant negative emotionality in a sex-dependent manner: high prenatal cortisol was associated with increased negative emotionality in females, and decreased negative emotionality in males. This study aims to test for this sex-specific effect in a different cohort, and investigate whether sex differences in fetal programming may be specific to glucocorticoid mechanisms by also examining a maternal salivary alpha-amylase (sAA) by sex interaction.

**Methods:**

88 pregnant women (mean gestational age = 27.4 weeks, SD = 7.4) collected saliva samples at home over two working days to be assayed for the hormone cortisol (range = 0.13–88.22 nmol/l) and the enzyme alpha-amylase (range = 4.57–554.8 units/ml). Samples were collected at waking, 30-min post-waking and 12 h post-waking. Two months after birth participants reported infant negative emotionality using the distress to limits subscale of the Infant Behavior Questionnaire.

**Results:**

The interaction between maternal prenatal cortisol and infant sex to predict distress to limits approached significance (p = 0.067). In line with our previous finding there was a positive association between prenatal cortisol and negative emotionality in females, and a negative association in males. The interaction between sAA and sex to predict distress was significant (p = 0.025), and the direction of effect was the same as for the cortisol data; high sAA associated with increased negative emotionality in females and reduced negative emotionality in males.

**Conclusions:**

In line with our previous findings, this research adds to an emerging body of literature, which suggests that fetal programming mechanisms may be sex-dependent. This is the first study to demonstrate that maternal prenatal sAA may be an important biomarker for infant behavior, and the findings have implications for understanding sex differences in developmental psychopathology.

## Highlights

• Effects of prenatal stress on fetal development may be mediated via glucocorticoids.
• We report that associations of prenatal cortisol with infant behavior are sex-specific.
• High maternal prenatal cortisol predicts increased negative emotionality in females.
• Alternatively, high cortisol predicts reduced negative emotionality in males.
• The same sex-interaction is evident for maternal salivary alpha-amylase.

## 1 Introduction

Prenatal psychological stress, which includes depression and anxiety, increases risk for adverse offspring outcomes, such as: preterm birth (Class et al., 2011), low birth weight (Zhu et al., 2010), behavioral difficulties (O’Connor et al., 2002, O’Connor et al., 2003; Connor et al., 2002, 2003), and psychiatric problems (Pearson et al., 2013, Van den Bergh et al., 2008). The prevailing mechanistic theory in perinatal psychiatry is that prenatal stress exerts influence on fetal developmental trajectories via glucocorticoid mechanisms. The animal literature has consistently supported this hypothesis (Barbazanges et al., 1996, Koehl et al., 1999, Lemaire et al., 2000, Maccari et al., 1995), however human studies have been less consistent. For example, evidence that prenatal stress is associated with increased maternal cortisol is mixed, with some studies supporting this association (Giesbrecht et al., 2012, Murphy et al., 2014, O’Connor et al., 2013, Obel et al., 2005) and others not (Braithwaite et al., 2016, Hellgren et al., 2013, Pluess et al., 2010). Similarly, evidence for associations between prenatal cortisol and offspring negative emotionality has been mixed, with evidence both for (Baibazarova et al., 2013, Davis et al., 2007) and against (Gutteling et al., 2005a) an association. Furthermore, there is a lack of evidence supporting a mediating role of maternal cortisol in associations between prenatal stress and adverse offspring outcomes; often only maternal cortisol or mood is reported to be associated with offspring outcomes (Davis and Sandman, 2010, Gutteling et al., 2005b, Sarkar et al., 2008).

One possible explanation for the disparate literature is that effects of prenatal stress may be sex-dependent. Sex differences in offspring outcomes following exposure to prenatal risks have been described in the animal and human literature. In animal studies, prenatal stress is associated with offspring depression and anxiety behaviors (Frye and Wawrzycki, 2003, Schulz et al., 2011, Zagron and Weinstock, 2006). Notably, these behaviors are present in the female, but not male, offspring. Interestingly, adrenalectomy of pregnant dams eliminated effects of prenatal stress on adverse female behavior (Zagron and Weinstock, 2006), further supporting sex-dependent effects mediated by glucocorticoid mechanisms. There is accumulating evidence in the human literature that prenatal risks for developmental psychopathology may be sex-dependent. For example, a range of prenatal risks, such as; stress, smoking and low birth weight, are associated with internalizing symptoms in females (Costello et al., 2007, Van den Bergh et al., 2008, Van Lieshout and Boylan, 2010) and externalizing symptoms in males (Li et al., 2010, Rodriguez and Bohlin, 2005). Prenatal anxiety has been linked to dampened diurnal cortisol release and depression in female offspring (Van den Bergh et al., 2008), and also gender-specific effects on vagal withdrawal during childhood (Tibu et al., 2014). Further, heightened cortisol in pregnancy has been linked with a range of other effects in female, but not male, offspring, including: a more difficult temperament (Sandman et al., 2013); increased amygdala volume (Buss et al., 2012); and anxiety and affective problems (Buss et al., 2012, Sandman et al., 2013). In addition, we have recently shown that heightened prenatal cortisol was associated with increased negative emotionality in female infants, but decreased negative emotionality in male infants at 5 weeks of age (Braithwaite et al., 2017). This literature supports an emerging concept that there may be sex-dependent processes underpinning effects of prenatal stress on developmental trajectories, whereby females become more reactive to challenge and more anxious, and males become less reactive and more aggressive (Glover and Hill, 2012, Sandman et al., 2013).

An explanation for difficulties in characterizing the role of glucocorticoids in associations between prenatal stress and adverse offspring outcomes is that other mechanisms may also be important. For example, changes in maternal sympathetic nervous system (SNS) activity may be an alternative pathway by which prenatal mood impacts fetal development (Braithwaite et al., 2014, Talge et al., 2007). The SNS is activated during psychological distress, resulting in increased noradrenaline levels. Noradrenaline does not cross the placental barrier (Giannakoulopoulos et al., 1999), however could indirectly influence fetal development by initiating vasoconstriction and reducing uterine blood flow. This mechanism could contribute to reduced birth weight or premature birth, both of which are associated with prenatal stress. Fluctuating oxygen and nutrient supplies to the developing fetus could also increase risk for psychological difficulties (Morsing et al., 2011). Animal studies show that both acute stress and intravenous noradrenaline induce decreased uterine blood flow (Shnider et al., 1979, Stevens and Lumbers, 1995). Initial human studies mirrored these findings (Sjostrom et al., 1997, Teixeira et al., 1999); however there have been a number of non-replications (Harville et al., 2008, Kent et al., 2002, Mendelson et al., 2011, Monk et al., 2012). The disparate findings could be attributable to difficulties in assessing uterine blood flow in a controlled laboratory setting, or could be explained by fetal sex-differences. An alternative method to assess SNS function is via the salivary biomarker, alpha-amylase. Salivary alpha amylase (sAA) is an enzyme produced by the salivary glands, which is controlled by SNS innervation. Increased sAA concentrations are evident during periods of psychological distress (Bosch et al., 1996, Chatterton et al., 1997, Skosnik et al., 2000), and sAA levels are inflated in people with depression (Ishitobi et al., 2010, Tanaka et al., 2012, Veen et al., 2013). In pregnant populations, heightened sAA has been associated with anxiety (Giesbrecht et al., 2013) and depression (Braithwaite et al., 2015b). It is currently unknown, however, whether prenatal sAA is an important biomarker for offspring development, and whether any effects may be gender-specific.

The primary aim of this study is to test if our recent finding, that high prenatal cortisol predicts increased negative emotionality in females, and decreased emotionality in males, is evident in a different cohort. A second aim is to investigate whether sex-dependent associations of the same kind may be specific to glucocorticoid mechanisms, by also testing for a SNS effect (sAA by gender interaction). Data used in this analysis has been previously published (Braithwaite et al., 2016, Braithwaite et al., 2015b), however in this manuscript we present a reanalysis of the data to specifically address the question of fetal sex.

## 2 Methods

### 2.1 Participants

Participants were a community sample of 103 first-time mothers and their infants participating in a longitudinal study based in Oxford, UK, designed to investigate the effects of prenatal mood disturbance on maternal and infant stress responses (Braithwaite et al., 2016, Braithwaite et al., 2015b). All participants were primiparous, more than 14 weeks pregnant, had a singleton pregnancy, were over the age of 18, had no medical complications associated with their pregnancy and were not currently taking any steroid-based medications. 10 participants reported medication use during pregnancy. The medications included: tri-cyclic anti-depressants (n = 2), selective serotonin re-uptake inhibitors (n = 1), ranitidine hydrochloride (n = 2) and omeprazole (n = 1) to treat gastro-esophageal reflux, antibiotics (n = 1), lactulose (n = 2) and thyroxine (n = 1). This research study was reviewed and approved by the Research Ethics Committee South Central Oxford B (REF: 12/SC/0473), and all participants provided informed consent for themselves and their infants to be included in the study. Complete prenatal and postnatal data was available for 88 mothers and their infants (39 males and 49 females), who comprise the sample for this analysis.

### 2.2 Procedure

This study comprised one prenatal and one postnatal assessment, which are detailed below.

#### 2.2.1 Prenatal assessment

At the time of the prenatal assessment participants were in either the second or third trimester of pregnancy (range = 106–281 days gestation, mean = 191.4 days, SD = 50.6). Participants were invited to a prenatal test session, which took place either at the Department of Psychiatry, University of Oxford, or at the participants’ home. This session took place between the hours of 1pm and 7pm, and lasted for approximately 90 min. Participants were asked to complete a questionnaire, which included questions about their demographic characteristics and current levels of depressive symptoms, and participated in a task, which has been described previously (Braithwaite et al., 2016). Participants were then asked to collect six saliva samples at home over two working days (3 per day), to be assayed for the hormone cortisol and the enzyme alpha-amylase. Samples were collected using the passive drool method, and participants were provided with six 2 ml cryovials and six saliva collection aids, as well as a stamped-addressed envelope to return the samples. On each day, samples were collected immediately after awakening, and 30 min and 12 h post-awakening. Participants were asked to refrain from eating, drinking, smoking and exercising for 30 min before each sample was collected. Participants stored the samples in their home fridges at ∼4 °C, before returning them to the Department of Psychiatry. Samples were shipped at room temperature, and remained at room temperature for a maximum of 24 h before being frozen at −20 °C at the Department of Psychiatry on arrival.

#### 2.2.2 Postnatal assessment

Participants and their infants were visited at home approximately 2 months (mean = 8.7 weeks, SD = 1.8) after they had given birth. Mothers reported postnatal symptoms of depression and completed a questionnaire about their infant’s behavior.

### 2.3 Measures

#### 2.3.1 Maternal prenatal and postnatal depression

Maternal prenatal and postnatal depressive symptoms were self-reported using the Edinburgh Postnatal Depression Scale (EPDS). The EPDS is the most widely used self-report questionnaire to identify symptoms of depression during the perinatal period. The scale consists of 10 items that describe common symptoms of depression, each item is scored from 0 to 3, and the scale has a maximum score of 30. A score of 13 or over is indicative of clinical levels of depression (Cox et al., 1987).

#### 2.3.2 Salivary cortisol and alpha-amylase

Salivary cortisol concentrations were quantified using an enzyme immunoassay kit (Salimetrics, UK) and the analysis was conducted in accordance with the manufacturer’s instructions. Samples were analyzed in singlet’s, and the minimum detectible concentration was 0.2 nmol/l when a 0.1 ml volume was assayed (inter-assay coefficient of variance = 7.75). Cortisol outliers that were more than three standard deviations from the mean were excluded (2 of 528 data points excluded).

Salivary alpha-amylase kits were sourced from Salimetrics, UK. The protocol used to determine salivary alpha-amylase concentration differed slightly from that recommended by the manufacturer, and this has been described previously (Braithwaite et al., 2015b). Following optimization of the assay, this method was tested for reliability and found to be highly replicable (intra-assay coefficient of variance = 3.73, inter-assay coefficient of variance = 9.55).

Cortisol and alpha-amylase have the opposite diurnal profile in pregnant and non-pregnant populations: diurnal cortisol is indexed by a sharp increase in concentration after awakening, followed by a gradual diurnal decline (Harville et al., 2007). Alternatively, there is a rapid decrease in alpha-amylase concentrations after awakening, followed by a gradual daily incline (Giesbrecht et al., 2013). Thus, in order to provide comparability of measures on both we used the log of the area under the curve (LogAUC) of the cortisol and alpha-amylase measures as an index of diurnal cortisol/alpha-amylase release. The area under the curve was calculated using the trapezoid method with respect to ground, taking the mean of the awakening, 30 min and 12 h post-awakening measures over two days. LogAUC cortisol/alpha-amylase was used as a main predictor in analyses.

#### 2.3.3 Infant behavior

At the 2-month postnatal visit, mothers reported their infant’s level of behavioral reactivity using the distress to limitations subscale of the Infant Behavior Questionnaire (IBQ) (Gartstein and Rothbart, 2003). The distress to limits subscale is one of three subscales of the IBQ used to create a composite IBQ score for negative emotionality (the others being the sadness and fear subscales). However, the fear and sadness subscales were not included in this study in order to reduce participant burden, and the scales are highly correlated (Gartstein and Rothbart, 2003). Mothers were required to report how often their infant engaged in various behaviors during the past week using a 7-point Likert scale from ‘Never’ to ‘Always’ (Gartstein and Rothbart, 2003).

### 2.4 Statistical analysis

The demographic characteristics of the male and female infants in the sample were assessed separately, and the two groups were compared using T-tests and chi-squared tests. Pearson’s bivariate correlations were used to assess associations between demographic variables, and the prenatal biological measures of stress. Separate linear regression models were constructed to examine the validity of prenatal cortisol and alpha-amylase, in interaction with gender, to predict infant distress to limitations. Maternal gestation and postnatal depression were included as confounders in all the regression models. We also considered the following confounders in initial analyses: maternal education, postnatal depression, maternal age, infant age, infant birth weight and maternal prenatal alcohol intake. However, none of these confounders were significant predictors of distress to limits (all p’s > 0.05), and therefore were not included in the main regression analyses. In the first model, infant gender and LogAUC cortisol/alpha-amylase were entered as main predictors, as was a gender*LogAUC cortisol/alpha-amylase interaction. The models were then re-constructed separately for male and female infants. In our previous publication we reported that the maternal waking cortisol measure specifically predicted infant negative emotionality in a sex-depended manner, whereas the measures taken at 30 min post-awakening and during the evening did not (Braithwaite et al., 2017). In the current paper we also examined whether this pattern of associations was similar for the cortisol data, and we conducted an exploratory analyses to investigate whether the same time-of-day effects may be evident in the sAA data. Three participants were using psychotropic mediation during pregnancy, therefore all analyses were repeated with these participants excluded (N = 85).

## 3 Results

### 3.1 Demographic characteristics and correlations

Demographic characteristics of the sample by gender are displayed in Table 1. Mothers of male infants had slightly higher levels of pre and postnatal depression, and higher levels of prenatal cortisol and alpha-amylase, however the difference between the groups was not statistically significant (all p’s > 0.05). Mothers of male infants also reported that their infants displayed slightly higher levels of distress to limits, but again this was not statistically significant. Maternal prenatal depression was not correlated with the LogAUC of either cortisol or alpha-amylase (p’s > 0.05), and maternal LogAUC cortisol and alpha-amylase were not correlated with each other (r = 0.081, p = 0.614). Infant distress to limits was not associated with prenatal depression (r = 0.073, p = 0.616), however there was a positive correlation between maternal postnatal depression and infant distress to limits (r = 0.240, p = 0.024), indicating that greater postnatal depression was associated with greater distress. Further examination revealed that this effect was specific to female infants (r = 0.295, p = 0.040); the association was non-significant for male infants (r = 0.159, p = 0.332), although in the same direction. Neither maternal LogAUC cortisol nor logAUC alpha-amylase was correlated with infant distress (r = 0.171, p = 0.239 and r = 0.065, p = 0.601 respectively).

**Table 1:** Demographic characteristics of the sample by gender.

	Whole sample (N = 88)	Males (n = 39)	Females (n = 49)
Maternal Variables			
Maternal age (m, SD)	31.2, 4.45	31.41, 4.07	32.45, 4.72
Maternal Education (n, %)			
NVQ	5 (5.7)	4 (10.3)	1 (2)
A level	3 (3.4)	3 (7.7)	–
Undergraduate degree	32 (36.4)	15 (38.5)	17 (34.7)
Postgraduate degree	48 (54.5)	17 (43.6)	31 (63.3)
Ethnicity (n, %)			
Caucasian	80 (90.9)	37 (94.9)	43 (87.7)
Black	1 (1.1)	–	1 (2)
Asian	4 (4.5)	1 (2.6)	3 (6.1)
Chinese	2 (2.3)	–	2 (4.1)
Mixed Race	1 (1.1)	1 (2.6)	–
Average units of alcohol/week (m, SD)	1.16, 0.37	1.23, 0.43	1.10, 0.31
Prenatal EPDS score (m, SD)	6.20, 5.09	6.84, 5.74	5.69, 4.49
Postnatal EPDS score (m, SD)	7.02, 4.05	7.38, 4.18	6.73, 3.99
LogAUC Cortisol (m, SD)	16.69, 5.19	17.10, 4.38	16.38, 5.79
LogAUC Alpha-amylase (m,SD)	43.21, 5.60	43.57, 4.77	42.98, 6.15

Infant Variables			
Birth weight, kg (m, SD)	3.42, 0.47	3.52, 0.51	3.32, 0.42
Gestational age at birth, weeks (m, SD)	40.14, 1.15	40.23, 1.03	40.08, 1.24
Age, weeks (m, SD)	8.67, 1.81	8.62, 1.44	8.71, 2.09
Distress to limitations (m, SD)	4.15, 0.75	4.21, 0.71	4.10, 0.79

### 3.2 Maternal prenatal cortisol and infant distress to limitations

The first regression model included a maternal prenatal cortisol by infant gender interaction term, to predict infant distress to limits. The point in gestation when saliva was sampled was not a significant predictor of infant distress to limits, but maternal postnatal depression was (Beta = 0.287, p = 0.010). Neither maternal LogAUC cortisol nor infant gender was a significant predictor of infant distress to limits at 2 months of age (Beta = −0.038, p = 0.781 and Beta = −0.013, p = 0.906 respectively). However, the LogAUC*gender interaction term approached significance (Beta = 0.212, p = 0.073). Male and female infants were then considered separately. Maternal prenatal cortisol did not reach significance in the prediction of infant distress to limits in either male (Beta = −0.279, p = 0.194) or female (Beta = 0.218, p = 0.167) infants. However, and notably, the direction of effect is opposite for male and female infants: a negative regression coefficient in males and a positive regression coefficient in females.

We then tested awakening cortisol in interaction with gender to predict infant distress. In line with the above, and our previous findings, the interaction term was significant (p = 0.047), and when considered separately there was a negative regression coefficient for males (Beta = −0.284, p = 0.095), and a positive coefficient for females (Beta = 0.115, p = 0.492). Also in line with our previous report, the association between maternal cortisol collected 30 min after awakening and during the evening in interaction with gender did not approach significance in the prediction of distress to limits (p = 0.474 and 0.195 respectively).

### 3.3 Maternal prenatal alpha-amylase and infant distress to limitations

As with the cortisol analysis, neither maternal prenatal LogAUC alpha-amylase nor infant gender significantly predicted infant distress to limits at 2 months of age (p = 0.565 and p = 0.922 respectively). However, the interaction between LogAUC alpha-amylase and gender was significant (Beta = 0.265, p = 0.039). When male and female infants were considered separately, the results were very similar to the cortisol analysis. In males, there was a negative association between prenatal alpha-amylase and infant distress to limits, which approached significance (Beta = −0.360, p = 0.072), indicating higher prenatal alpha-amylase is associated with lower levels of distress. Conversely, in female infants there was a positive association between prenatal alpha-amylase and distress to limitations (Beta = 0.157, p = 0.257), signifying that higher prenatal alpha-amylase was associated with greater distress, although the effect was non-significant.

We also conducted exploratory analyses to investigate time-of-day effects in the sAA data, as we did for the cortisol data. There was no interaction between sAA collected on awakening and during the evening with infant sex (p’s = 0.111 and 0.151 respectively). However, there was a significant interaction between the sAA measure taken 30 min after waking and infant sex (Beta = 0.332, p = 0.004). When males and females were considered separately, there was a significant effect of maternal sAA for both males and females, and the effects were in the opposite direction (males: Beta = −0.396, p = 0.044, females: Beta = 0.264, p = 0.044).

All analyses were repeated with the three participants who were using psychotropic medication excluded, however this did not change the results (data not shown).

## 4 Discussion

This study set out to examine sex-specific effects of maternal prenatal biomarkers of stress on infant negative emotionality. We found that an interaction between prenatal LogAUC cortisol and infant gender approached significance, and when males and females were considered separately, effects of prenatal cortisol were in opposite directions, but did not reach significance. In line with our previous findings from a different cohort (Braithwaite et al., 2017), high prenatal cortisol was positively associated with distress to limits in females, and negatively associated with distress to limits in males. Also in line with our previous analyses, there was a significant interaction between the waking cortisol measure and infant sex to predict distress to limits, however this interaction did not reach significance when using the cortisol measure taken 30 min after waking or during the evening. It is unclear why waking cortisol may be particularly relevant to infant behavior, however research focused on prenatal cortisol and obstetric outcomes highlight that various indices of morning cortisol more strongly predict obstetric outcomes than measured taken throughout the day (Entringer et al., 2011, Goedhart et al., 2010, Kivlighan et al., 2008). We also found a significant interaction between prenatal LogAUC sAA and infant gender in the prediction of infant distress to limits, and again, the direction of effect was opposite for male and female infants, but did not reach statistical significance. High prenatal sAA was associated with increased distress to limits in females, and decreased distress to limits in males. To our knowledge, this is the first study to show that maternal prenatal sAA may be an important biomarker for infant behavioral outcomes. Exploratory analyses revealed a significant interaction between the sAA measure taken 30 min after waking and infant sex to predict distress to limits, however, this interaction was non-significant when using the waking and evening sAA measures. It is also unclear why the timing of the sAA measure may be relevant for infant behavior, and this finding requires replication in a different cohort before its significance is discussed.

The prevailing mechanistic theory in perinatal psychiatry is that effects of maternal prenatal stress on adverse offspring outcomes are mediated by glucocorticoid mechanisms. The animal literature has been robust in supporting this theory, however the human literature is less consistent. Previously, we have shown that maternal prenatal cortisol predicted infant negative emotionality in a sex-dependent way (Braithwaite et al., 2017), and the current study tested whether this effect might be specific to maternal cortisol. However, we found that maternal prenatal sAA also predicted infant negative emotionality in the same sex-dependent manner. It is possible that effects of maternal prenatal stress on offspring outcomes are mediated exclusively via glucocorticoid mechanisms, however alternatives are infrequently considered. It is therefore possible that the variable often measured (i.e. maternal cortisol) may not be part of the causal mechanism. There is a paucity of data directly demonstrating mediation effects of maternal prenatal cortisol on offspring outcomes, and often associations with offspring outcomes are reported only for maternal mood, or maternal cortisol separately (Davis and Sandman, 2010, Gutteling et al., 2005b, Sarkar et al., 2008). It is not possible to test maternal cortisol and sAA as causal predictors using observational data, but it is an interesting question for future experimental designs such as randomized controlled-trials and experimental animal models.

The results reported in this study also have implications for our understanding of sex differences in developmental psychopathology. During pre-adolescence there is a predominance of attention and conduct disorders in boys, however post-puberty there is a predominance of female affective disorders. The etiologies of these sex differences are poorly understood, and are likely explained to some extent by differential risk exposure for male and female infants (Moffitt et al., 2001). However, there are two further possibilities to explain sex differences in developmental psychopathology that have received less attention. First, in the context of prenatal stress it is possible that risks for psychopathology are different in males and females. For example, low birth weight and prenatal stress have been associated with internalizing symptoms in 2.5-year-old girls (Sharp et al., 2015) and adolescent depression in females, but not males (Costello et al., 2007, Quarini et al., 2016, Van den Bergh et al., 2008). Our current findings are consistent with this hypothesis, as we have shown that raised prenatal cortisol and sAA are positively associated with negative emotionality in girls, which is an early marker of poor social competence and psychopathology (Degnan et al., 2008, Kopp, 1989).

A second alternative is that the risks for psychopathology are the same for males and females, but the biological mechanisms leading to the onset of psychopathology are different. A good example of this comes from the vagal reactivity literature. An early study demonstrated that high vagal tone was associated with improved social competence and emotion regulation in boys, but with poorer functioning in girls (Eisenberg et al., 1995). Recent studies have replicated this finding, with higher vagal tone or vagal withdrawal being consistently associated with better functioning in boys and, critically, poorer functioning in girls (Hinnant and El-Sheikh, 2013, Morales et al., 2015). It may be that the opposite changes in vagal reactivity following prenatal adversity are on the causal pathway to different psychiatric outcomes for males and females. There is initial evidence to support this hypothesis: prenatal anxiety and low birth weight have opposite effects on vagal reactivity in males and females (Tibu et al., 2014). Greater vagal reactivity has also been shown to predict externalizing and oppositional defiant behaviors in a sex-dependent way (Morales et al., 2015; Vidal-Ribas et al., in press). Results from the current study, alongside our previous findings (Braithwaite et al., 2017), provide initial evidence to suggest that prenatal stress may have similar sex-dependent effects on programming of glucocorticoid and SNS mechanisms. We have reported, using data from two different cohorts, an increase in negative emotionality in girls, and a decrease in negative emotionality in boys, following high prenatal cortisol or sAA exposure. Negative emotionality in infancy has been related to noncompliance (Stifter et al., 1999), aggressive behavior (Crockenberg et al., 2008) and poor emotion regulation (Calkins et al., 2002) in childhood. Thus, following exposure to high prenatal stress biomarkers, females may be at increased risk of these outcomes, whereas reduced negative emotionality in males may represent reduced risk, or a protective mechanism. Alternatively, low emotionality may lead to certain forms of aggression in males, such as those associated with callous unemotional traits (Frick and White, 2008).

The full extent of the *in utero* pathways leading to sex-dependent biological mechanisms of effect remains to be elucidated. There is evidence from animal and human research that the pathway from elevated maternal cortisol to increased negative emotionality in females may be via HPA programming. In animal models exposure to prenatal stress or elevated maternal glucocorticoids results in altered hippocampal and hypothalamic glucocorticoid receptor expression (Szuran et al., 2000, Weinstock et al., 1992), heightened HPA activity (McCormick et al., 1995, Szuran et al., 2000, Weinstock et al., 1992) and a depressive/anxious phenotype in female offspring (Frye and Wawrzycki, 2003, Schulz et al., 2011, Zagron and Weinstock, 2006). Similarly, the human literature reports heightened HPA function (Van den Bergh et al., 2008), increased amygdala activity (Buss et al., 2012), and depressive/anxious symptoms (Buss et al., 2012, Sandman et al., 2013) in females exposed to maternal prenatal psychological distress. It is unclear, however, what *in utero* biological mechanisms may lead to reduced emotionality in males. Animal research suggests that prenatal stress leading to learning deficits in males and anxious behavior in females may be underpinned by a sex-depended reduction in neurogenesis and dendritic morphology in the prefrontal cortex and hippocampus (Weinstock, 2011). There is also some initial evidence to suggest that there may be opposite effects on intracellular signaling (Bangasser et al., 2010), receptor trafficking (Bangasser et al., 2010), and epigenetic regulation of the HPA axis in males and females in the context of prenatal stress (Braithwaite et al., 2015a), which may contribute to changes in emotionality. Further understanding of sex-specific *in utero* biological mechanisms, which may increase risk for later psychopathology, is a key area for future research.

This study has a number of strengths, including the prospective longitudinal design, and validated measures of maternal depression and salivary biomarkers. However, there are a number of limitations that should be considered. The small sample size is a key limitation, as a larger sample would have the statistical power to detect smaller effects. In some instances where there were significant interaction terms involving infant sex, the separate effects in males and females were non-significant. These were all in the predicted direction, consistent with true effects that were non-significant because of the reduced statistical power in the subgroups. Equally, they may have arisen by chance with a likelihood represented by the value of P. Second, we only assessed maternal stress biomarkers once in pregnancy, during either the second or third trimester. Had we measured biomarkers during each trimester, we would have been able to test for timing effects of raised prenatal biomarkers on infant behavior. We did not assess maternal compliance with the saliva sampling procedure; therefore alterations in the timing of saliva collection may have introduced error into our analyses. However, we did see the expected diurnal patterns in salivary cortisol and alpha-amylase release, suggesting that, in the main, participants complied with the saliva sampling protocol. Our measure of infant distress to limits was based on maternal report, and may therefore be subject to maternal postnatal mood, bias and miss-reporting. That being said, effects were evident when controlling for postnatal depression in the model. A more robust method to assess infant behavior is via video-observation; our previous paper to report a sex by prenatal cortisol interaction in the predication of infant negative emotionality assessed infant behavior in this way (Braithwaite et al., 2017). Furthermore, we did not include the fear and sadness subscales of the IBQ, which are used to compose an IBQ score for negative emotionality. Finally, the IBQ was designed to measure infant behavior from the age of three months, however the infants in this study were on average two months old when the IBQ was administered, and may therefore not be an appropriate measure of infant behavior. However, the distress to limits subscale of the IBQ has shown stability from 2 weeks to 12 months of age (Worobey and Blajda, 1989).

### 4.1 Conclusion

To conclude, we found evidence to suggest that prenatal salivary cortisol and alpha amylase are associated with infant negative emotionality in a sex-dependent way. Our findings add to an emerging body of literature, which suggests that there may be sex differences in effects of prenatal stress on offspring outcomes. This finding also has implications for further understanding sex differences in developmental psychopathology, which is important when designing targeted intervention and prevention strategies.

## Conflicts of interest

SEM has received consultancy payments from p1Vital, grant income from J&J and has participated in paid speaking engagements for Lilly UK. All other authors report no conflicts of interest.
